# Self-assembling viral histones are evolutionary intermediates between archaeal and eukaryotic nucleosomes

**DOI:** 10.1038/s41564-024-01707-9

**Published:** 2024-05-28

**Authors:** Nicholas A. T. Irwin, Thomas A. Richards

**Affiliations:** 1 Merton College, University of Oxford, Oxford, UK; 2 Department of Biology, University of Oxford, Oxford, UK; 3 Present Address: Gregor Mendel Institute (GMI), Austrian Academy of Sciences, Vienna BioCenter (VBC), Vienna, Austria

**Keywords:** Molecular evolution, Chromatin, Virology

## Abstract

Nucleosomes are DNA–protein complexes composed of histone proteins that form the basis of eukaryotic chromatin. The nucleosome was a key innovation during eukaryotic evolution, but its origin from histone homologues in Archaea remains unclear. Viral histone repeats, consisting of multiple histone paralogues within a single protein, may reflect an intermediate state. Here we examine the diversity of histones encoded by Nucleocytoviricota viruses. We identified 258 histones from 168 viral metagenomes with variable domain configurations including histone singlets, doublets, triplets and quadruplets, the latter comprising the four core histones arranged in series. Viral histone repeats branch phylogenetically between Archaea and eukaryotes and display intermediate functions in *Escherichia coli*, self-assembling into eukaryotic-like nucleosomes that stack into archaeal-like oligomers capable of impacting genomic activity and condensing DNA. Histone linkage also facilitates nucleosome formation, promoting eukaryotic histone assembly in *E. coli*. These data support the hypothesis that viral histone repeats originated in stem-eukaryotes and that nucleosome evolution proceeded through histone repeat intermediates.

## Abstract (web summary)

Phylogenetic and functional analyses suggest that viral histones were acquired from early eukaryotes during eukaryogenesis and that nucleosome evolution proceeded through histone repeat intermediates.

## Main

Nucleosomes are a core component of eukaryotic nuclei, forming the structural basis of chromatin and coordinating processes from gene expression to chromosome segregation. Composed of a DNA–protein complex consisting of the four individual histones, H2A, H2B, H3 and H4, the nucleosome and its associated functions were key innovations during eukaryotic evolution^1,2^. However, functional constraints and the seeming extinction of stem-eukaryotes have concealed how these dynamic systems evolved from simpler histone homologues in Archaea^3–6^.

Large double-stranded DNA viruses, such as the Nucleocytoviricota viruses (NCVs), are a promising source of additional gene family diversity as they can encode hundreds of co-opted genes with both recent and ancient cellular ancestries^7–10^. Indeed, histone proteins have been characterized from NCV genomes^11–13^. These histones are related to the eukaryotic histone paralogue families and exist as individual or repeated histone-fold domains, termed histone repeats. For example, H2B-H2A and H4-H3 doublets in marseilleviruses form eukaryotic-like nucleosomes essential for viral genome packaging^14–16^. Moreover, phylogenetic analyses place these histones between archaeal and eukaryotic homologues, prompting competing hypotheses about their origins^11,17^. In particular, whether these repeats, formed by post-hoc fusion between recently transferred eukaryotic histones, represent the viral progenitors of eukaryotic histones or were acquired following gene transfer from primordial eukaryotes remains unclear.

To assess these hypotheses, we examined the phylogenetic and functional diversity of viral histones. By surveying NCV metagenomes, we identified hundreds of histones with variable configurations, including histone quadruplets which comprise the four core histones arranged in series. Viral histone repeats branch between Archaea and eukaryotes in phylogenetic trees, and histone quadruplets exhibit intermediate functions in *E. coli*, self-assembling into eukaryotic-like nucleosomes capable of stacking into archaeal-like oligomers. The linkers conjoining histone repeats facilitate nucleosome formation and promote the assembly of eukaryotic histones in *E. coli*. Together, these data suggest that viral histone repeats represent molecular relics acquired by viruses from stem-eukaryotes during eukaryogenesis and point to an empirical hypothesis for the origin of the nucleosome.

## Results

### The diversity and distribution of viral histones

**Fig. 1:**
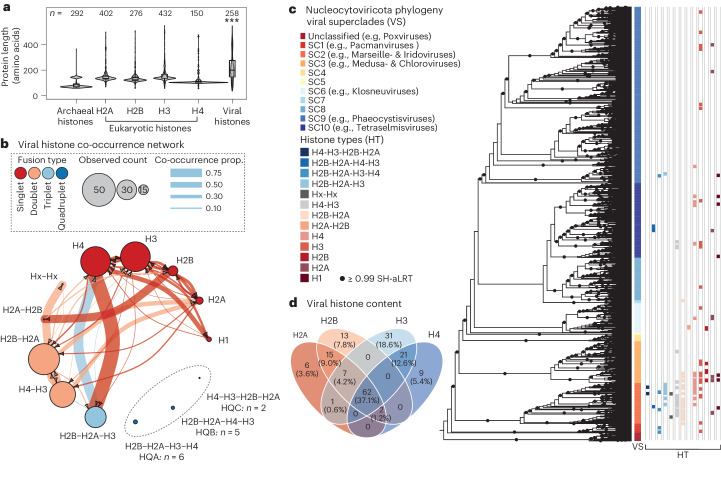
Viral histones are present and possess variable repeat domain architectures in diverse Nucleocytoviricota viruses. **a**, Amino acid length distributions of cellular and viral histones with sample sizes denoted. The centre line of the boxplots denotes the median and the upper and lower borders span from the first to the third quartiles, with whiskers extending 1.5 times the interquartile range. ***P = 8.47 × 10^−6^, *F* = 19.94, d.f. = 1 (contrast analysis of variance (ANOVA), cellular versus viral histones). **b**, A network showing the composition, frequency and co-occurrence proportions of each viral histone. **c**, Maximum-likelihood phylogeny of the Nucleocytoviricota based on five concatenated core viral genes (major capsid protein, B family DNA polymerase, packaging ATPase, a primase-helicase and a transcription factor) with the presence of different histone types (HT) and superclade taxonomy denoted. The phylogeny was re-run on the basis of the alignment of ref. ^7^ using the LG + F + R10 substitution model in IQ-Tree. **d**, A Venn diagram displaying histone complements in individual Nucleocytoviricota genomes.

**Extended Data Fig. 1:**
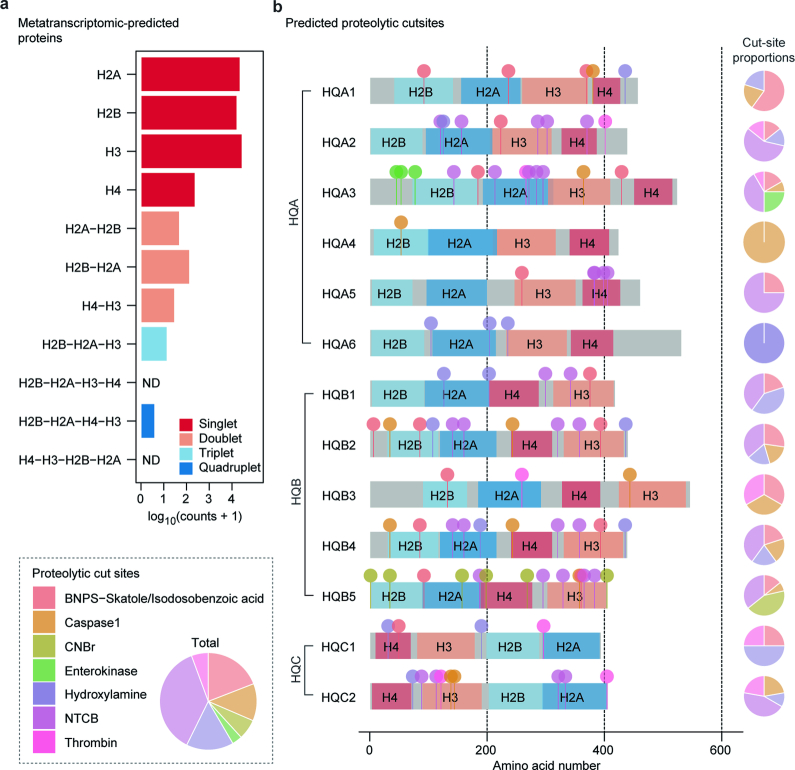
Transcription and predicted processing of viral histones indicates expression of complete repeat forms. **a**, Detection of histone repeats in metatranscriptomic data generated during the Tara Oceans Project. ND; not detected. Note that counts of individual histones will include eukaryote-derived transcripts. **b**, Predicted proteolytic cut-sites and diagrams of viral histone quadruplets. Cut-sites and cutter proportions are denoted and were predicted using ExPASy PeptideCutter. Source data

To characterize viral histone diversity, we surveyed the predicted proteomes of NCVs, viruses known to encode histone proteins^17^. Using profile hidden Markov models representing the core eukaryotic histone families and archaeal histones, we searched predicted proteins derived from both NCV genomes (*n* = 205) and assembled NCV metagenomes (*n* = 2,074)^7^. This approach identified 258 complete histone genes from 168 viruses. Viral histones had longer predicted coding sequences relative to cellular homologues due to the presence of histone repeats such as doublets (*n* = 90), triplets (*n* = 32) and quadruplets (*n* = 13), as noted previously^11,12,17^ (Fig. 1a,b). These histone repeats exhibited partially constrained domain orders, with H2A/H2B and H3/H4 nearly always in series (Fig. 1b). Similar histone repeats were also detected in ocean metatranscriptomes^18^ indicating that these genes are not artefactual predictions or immediately processed post-transcriptionally (Extended Data Fig. 1a). Likewise, consistent predicted post-translational cleavage sites between histone domains were absent in histone quadruplets (Extended Data Fig. 1b), although additional factors (for example, ribosome skipping) could facilitate individual histone translation. Together, these data indicate that viral histones can be encoded and possibly expressed as repeats.

**Extended Data Fig. 2:**
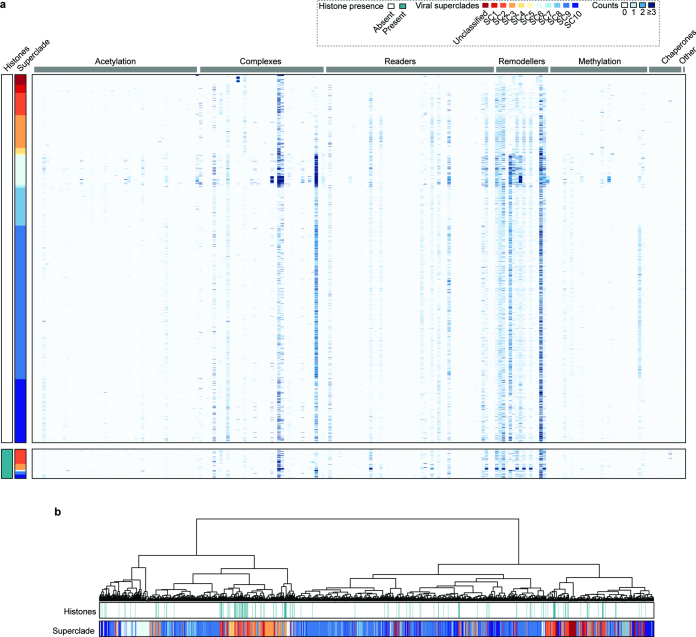
Chromatin machinery does not show a co-association with histones in Nucleocytoviricota genomes. **a**, A heatmap showing the prevalence of chromatin-associated proteins across viral genomes and metagenomes based on proteins curated by Grau-Bové et al.^50^ Histone presence and viral superclade classifications are denoted by coloured strips and protein annotations are available from FigShare. **b**, Hierarchical clustering of viral taxa based on chromatin machinery repertoires. Clustering was conducted using the Ward D2 method based on correlation distances.

Assessing histone distributions over the NCV phylogeny revealed that histone repeats were common in the early-branching superclades including marseilleviruses, iridoviruses and medusaviruses, whereas deeper-branching viral superclades mainly encoded histone singlets (Fig. 1c). Many viruses also encoded multiple histones, which was reaffirmed by co-occurrence analysis (Fig. 1b,d). Regardless of viral genome completeness, many histone-encoding viruses contained a full histone complement, achieved through combinations of histone types such as an H2B-H2A-H3 triplet and separate H4 singlet (Fig. 1b,d). Histone quadruplets were detected in three configurations (termed HQA, HQB and HQC), each providing a full complement, but never co-occurring with other histones (Fig. 1b). Histone composition was also constrained, as viruses rarely encoded an H3 or H4 in isolation with an H2A or H2B (Fig. 1d). Given these patterns, we investigated whether additional chromatin-related proteins were present in NCV genomes (Extended Data Fig. 2a). Although some putative chromatin-associated proteins were detected, there was no connection between their abundance and histone content (Extended Data Fig. 2b). This implies that although histone composition is important and constrained, viral histones probably either function autonomously or are supplemented by host-encoded factors.

### The phylogeny and evolution of viral histone repeats

**Fig. 2:**
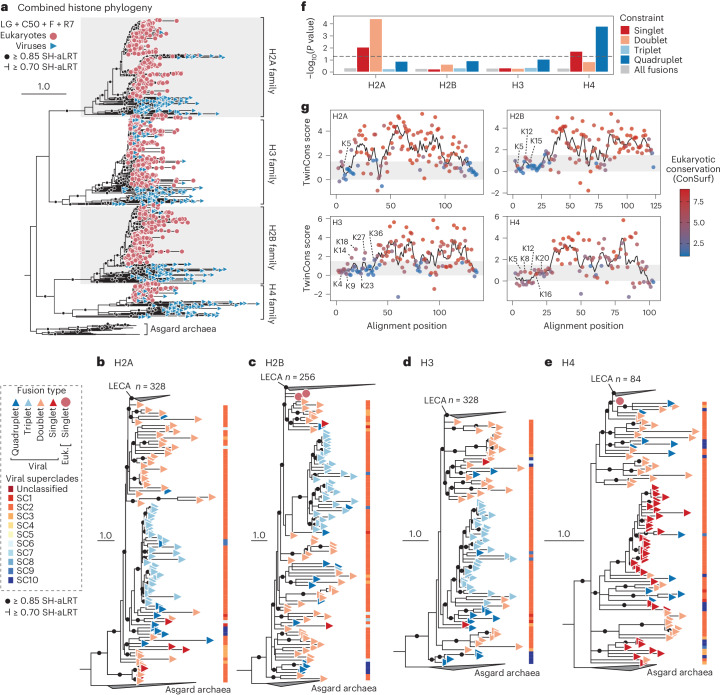
The phylogeny of viral histone repeats reveals their dynamic evolution and intermediate branching position between eukaryotes and Archaea. **a**, Maximum-likelihood (ML) phylogeny generated from an alignment of histone domains derived from eukaryotes, viruses and Asgard archaea. Statistical support was generated using SH-aLRT tests (*n* = 1,000). **b**–**e**, ML phylogenies for each of the core histone families including H2A (**b**, LG + R9), H2B (**c**, LG + R7), H3 (**d**, LG + R7) and H4 (**e**, LG + R5). Eukaryotic clades (including viral histones branching within eukaryotes) have been collapsed and the node corresponding to the last eukaryotic common ancestor (LECA) is labelled. Each tree was rooted with the same histone homologues from Asgard archaea (*n* = 10). For all phylogenies, nodes with support less than 0.70 SH-aLRT were collapsed. Scale bars represent the average number of substitutions per site. Full phylogenies can be viewed at https://itol.embl.de/shared/31JevmUKUPjD4. **f**, *P* values resulting from AU topology tests comparing best trees (**b**–**e**) to trees where the monophyly of different repeat types has been constrained. The dashed line denotes *P* = 0.05. Exact *P* values and likelihood scores are available in Extended Data Table 1. **g**, TwinCons analysis comparing conservation in histone domains between eukaryotes and viral proteins. Residues are coloured on the basis of eukaryotic histone site conservation determined using ConSurf. Dotted and dashed lines denote TwinCons values of zero and 1.5, respectively. Modifiable lysine residues are labelled for reference.

**Extended Data Fig. 3:**
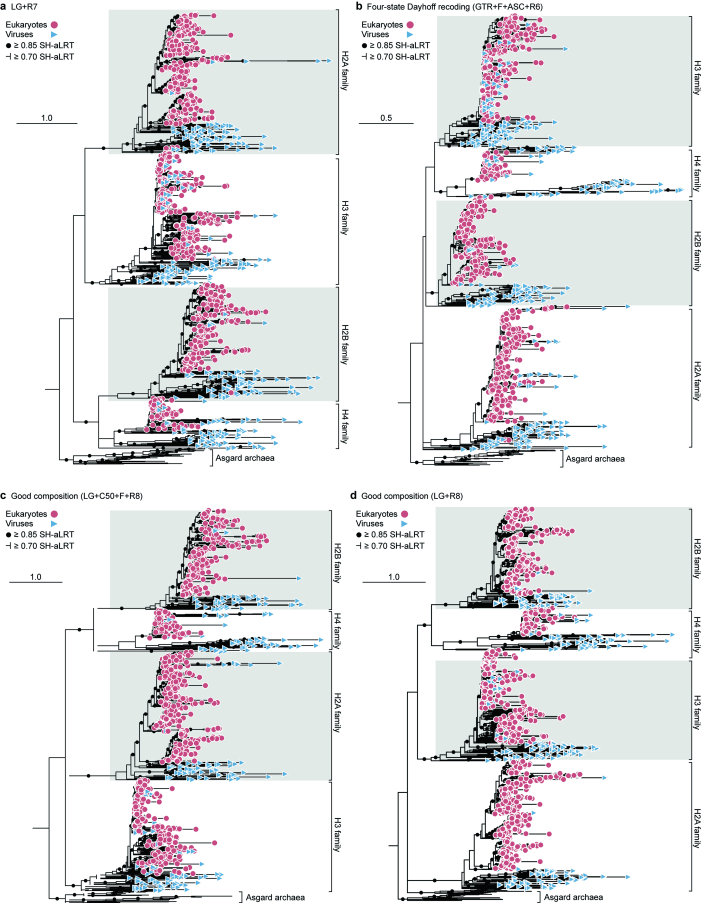
Histone phylogenies are robust to model selection but vary when accounting for compositional bias and after amino acid recoding. ML histone phylogenies generated using the same alignment as the tree in Fig. 2a but using alternative models (**a**), after four state-Dayhoff alignment recoding (**b**), and various models after removing compositionally biased sequences (**c**, **d**). Statistical support was generated using SH-aLRT (*n* = 1,000) and substitution models were selected using ModelFinder and are denoted in parentheses. Full phylogenies can be viewed at https://itol.embl.de/shared/2V0SeF3AOvgMe.

Given the abundance of viral histones, we next sought to evaluate their evolutionary history. Histone domains extracted from the repeats were analysed phylogenetically alongside eukaryotic and archaeal homologues. Using improved substitution models and histone sampling, we generated a well-resolved phylogeny for the entire histone superfamily, which placed histones H2A and H3, and H2B and H4 as sister clades (Fig. 2a). This fits with functional characteristics, such as the tendency for H2A and H3 to diverge into histone variants and the suggestion that H2A/H2B arose by duplication of H3/H4 precursors^1,4^. Yet although this topology was robust to model selection, the sisterhood of H2A and H3 was influenced by amino acid composition, the phylogeny was destabilized by amino acid recoding, and topology tests failed to reject alternative histone relationships (Extended Data Fig. 3 and Table 1). Unlike the overall topology, the branching of viral histones was robust and consistent with previous work^11,17^ (Fig. 2a and Extended Data Fig. 3). To account for long-branch attraction, we used varying subsets of sequences (Fig. 2a–e), multiple substitution models (Fig. 2 and Extended Data Fig. 3), repetitive tree searches (*n* = 100) to avoid local maxima, removed compositionally biased sequences and recoded the alignments using 4-state Dayhoff recoding (Extended Data Fig. 3). In each case, consistent tree topologies were recovered. On the basis of these phylogenies, viral histones were assigned to specific histone families, but repeat domains almost exclusively branched outside the eukaryotic clades, unlike those branching within eukaryotes which were mostly singlets (Fig. 2a–e). Accordingly, histone repeats seem to have an ancient ancestry whereas many viral histone singlets exemplify relatively recent horizontal gene transfers (HGT) from eukaryotes, similar to those observed in baculoviruses and polydnaviruses, as hypothesized previously^9,17^.

Examining individual histone families revealed the relatedness and evolution of viral histone repeats (Fig. 2b–e). Histone phylogenies are complicated by the time scales they cover and the limited information provided by their short, albeit highly conserved sequences. Nonetheless, these phylogenies highlighted the nesting of eukaryotic sequences within viral homologues and illustrate histone repeat dynamics. For example, histone quadruplets often branched closely with doublets, indicating repeated fission or fusion events. Similarly, the relatedness between histone quadruplets and triplets suggests that triplets evolved following the excision of H4 from a quadruplet, although the presence of a quadruplet within the triplet clade could imply reversibility (Fig. 2b–e). These observations were supported by topology testing, as H2A and H4 phylogenies rejected the monophyly of histone singlets, doublets and quadruplets, but the monophyly of all histone repeats could not be rejected and these trees often placed the repeats within the eukaryotic clade (Fig. 2f and Extended Data Table 1). Therefore, although these data show repeated fission and fusion during viral histone evolution, the exact order of events may be impossible to reconstruct.

**Extended Data Fig. 4:**
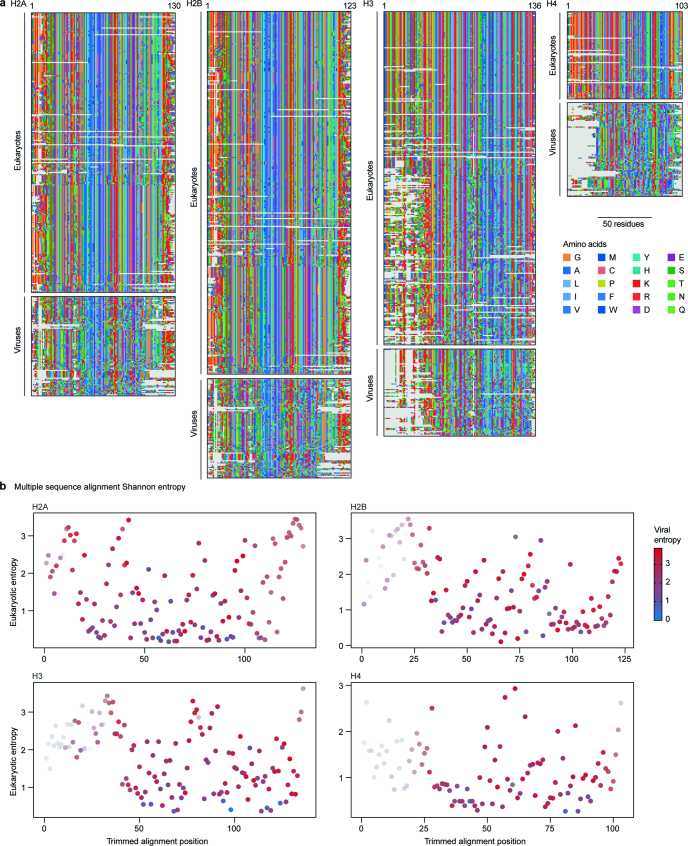
Multiple sequence alignments and Shannon entropy plots for eukaryotic histones and viral histone repeats. **a**, Trimmed multiple sequence alignments for histone H2A, H2B, H3, and H4 domains. Eukaryotic and viral histone repeats have been noted. Amino acid sites have been coloured by residue and gaps are represented by light grey boxes. **b**, Shannon entropy for each alignment site across the H2A, H2B, H3, and H4 alignments. The y-axis represents eukaryotic entropy whereas each point is coloured based on viral histone entropy. Point transparency reflects the proportion of gaps within the viral sequences.

**Extended Data Fig. 5:**
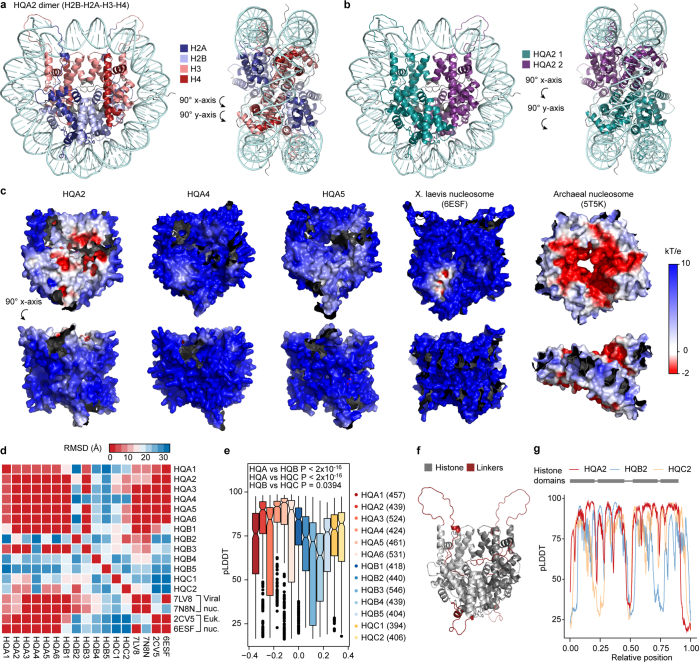
AlphaFold-predicted structures of histone quadruplet dimers reveal a capacity for nucleosome formation. The predicted structure of a histone quadruplet dimer using HQA2 as an example. Individual histone domains (**a**) or monomers (**b**) have been coloured and DNA from the structure of a *Xenopus laevis* nucleosome (PDB 6ESF) has been superimposed. **c**, Predicted structures of HQA2, HQA4, and HQA5 dimers alongside the experimentally determined structures of a eukaryotic and archaeal nucleosome. Protein surfaces have been coloured based on predicted charge. **d**, Heatmap depicting root mean square deviation (RMSD) values for noted histone structures. **e**, pLDDT (predicted local distance difference test) confidence metrics for individual residues in the predicted structures of each of the histone quadruplets. Statistical comparisons of the mean confidence values were made between quadruplet types using contrasted ANOVAs after Bonferroni correction. HQA vs HQB (*p* < 2 × 10^−16^, F = 96.637, df = 1), HQA vs HQC (*p* < 2 × 10^−16^, F = 189.030, df = 1), HQB vs HQC (*p* = 0.0394, F = 4.244, df = 1). The centre line of the boxplots denotes the median and the upper and lower borders span from the first to the third quartiles with whiskers extending 1.5 times the interquartile range. Sample sizes are denoted in parentheses. **f**, Predicted HQA2 structure with amino acid linkers highlighted in red. **g**, pLDDT confidence values across the structures of HQA2, HQB2, and HQC2. Decreases in confidence scores roughly correspond to linkers and histone domain turns. The approximate positions of the histone domains and linkers are shown. Note that the disordered C-terminus of HQA2 has been excluded from structure images.

To corroborate the phylogenies, we examined whether the evolutionary distinctiveness of viral histone repeats coincides with structural differences. To do this, we compared amino acid conservation between eukaryotic histone domains and the orthologous domains from the viral repeats (Fig. 2g and Extended Data Fig. 4). Using TwinCons^19^, we compared amino acid conservation between the two groups. In brief, values above 1.5 approximately represent conserved sites, values below zero indicate little conservation, and values in between denote divergence. In addition, we also analysed the conservation of eukaryotic histone residues using ConSurf^20^. This analysis revealed that the histone-fold is conserved in both eukaryotes and viral repeats, whereas the N and C termini were not. This included the divergence of key eukaryotic N-terminal sites which are post-translationally modified and influence genomic regulation, although some viral N termini contained modifiable lysines^21^. Viral histone repeats also exhibited increased Shannon entropy along the entire domain, consistent with elevated variability relative to eukaryotic histones (Extended Data Fig. 4b). To understand the consequences of this variation, we predicted the quaternary structures of histone quadruplets, as their lack of co-occurrence with other histones implies functional autonomy (Fig. 1b). Multimeric AlphaFold^22^ predictions indicated that histone quadruplets assemble into dimers, constituting pseudo-octameric structures with similarity to eukaryotic and viral nucleosomes (Extended Data Fig. 5a–d), although HQA had better defined structures based on predicted local distance difference tests (pLDDT) compared with HQB and HQC (Extended Data Fig. 5e). Individual histone domains were joined by disordered amino acid linkers (Extended Data Fig. 5f,g) which varied in length (median lengths and standard deviations: HQA, 12 ± 5, 56 ± 6, 16 ± 8, *n* = 6; HQB, 13 ± 1, 47 ± 8, 53 ± 3, *n* = 5; HQC, 38 ± 7, 23.5 ± 1, 13.5 ± 1, *n* = 2). Some of the quadruplets also featured disordered terminal extensions, but these were inconsistent and not conserved (Extended Data Fig. 1). This modelling suggests that histone quadruplets, similar to histone doublets^14,15^, have the capacity to form nucleosomes.

### Viral histones self-assemble into nucleosome-like structures

**Fig. 3:**
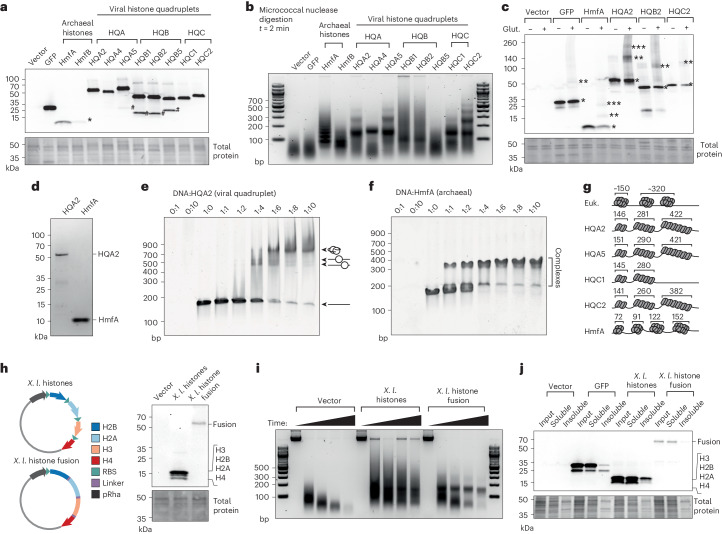
Viral histone quadruplets self-assemble into nucleosomal structures. **a**, Anti-6xHis immunoblot on whole-cell extracts from induced *E. coli*. Degradation products (#) and archaeal HmfB (*) are noted. **b**, Gel electrophoresis of genomic DNA from *E. coli* after 2 min of micrococcal nuclease (MNase) digestion. **c**, Anti-6xHis immunoblot of whole-cell extracts with and without glutaraldehyde crosslinking. Putative oligomers are highlighted with asterisks. Note that some higher-molecular-weight products are still recovered without crosslinking (for example, HQA2 and HQB2) despite denaturing conditions. **d**, Coomassie-stained SDS–PAGE gel showing purified viral HQA2 and archaeal HmfA. **e**,**f**, Electrophoretic mobility shift assays for HQA2 (**e**) and HmfA (**f**) in combination with 147 bp Widom 601 DNA. Protein to DNA molar ratios and diagrams of hypothesized complexes are noted. **g**, Hypothetical model for nucleosome formation with eukaryotic, archaeal and viral histones. **h**, Diagram of *Xenopus laevis* (*X. l*.) histone constructs and anti-6xHis immunoblot of whole-cell extracts from *E. coli* expressing each construct. RBS, ribosome binding site. **i**, Gel electrophoresis of genomic DNA from *E. coli* after 1, 2, 5 and 15 min of MNase digestion while expressing *X. laevis* histone vectors. **j**, Anti-6xHis immunoblot showing input, soluble and insoluble protein fractions from *E. coli* expressing GFP or *X. laevis* histones induced by 1 mM rhamnose. Each experiment was repeated at least twice independently with equivalent results. Units for molecular weight markers are noted for each figure: bp, base pair; kDa, kilodalton. Source data

**Extended Data Fig. 6:**
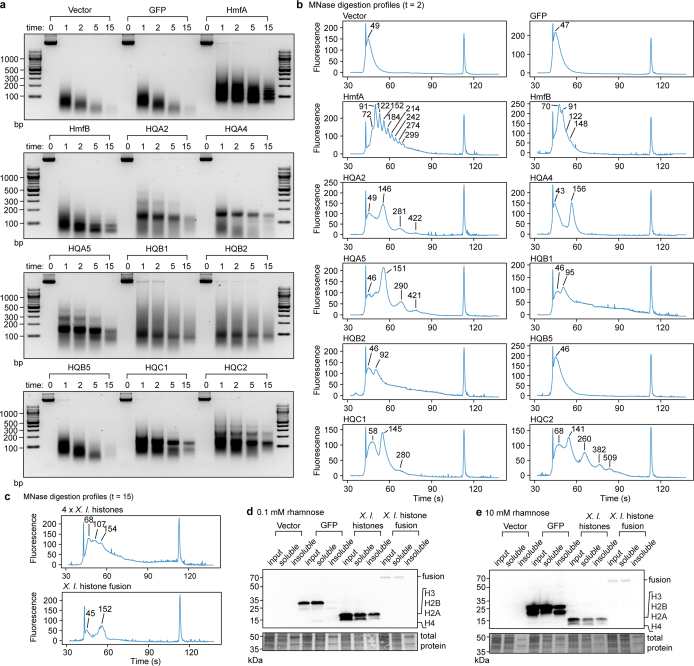
Micrococcal nuclease digestion profiles and the impact of histone fusion of protein solubility. **a**, Gel electrophoresis of genomic DNA from *E. coli* after 1, 2, 5, and 15 minutes of micrococcal nuclease (MNase) digestion during the expression of different vectors. **b**, BioAnalyzer size spectra for genomic DNA after two minutes of MNase digestion (see Fig. 2b). Peaks and their associated fragment sizes in base pairs are noted. **c**, BioAnalyzer size spectra for genomic DNA after 15 minutes of MNase digestion in the presence of individual or fused *X. laevis* histones. **d**, **e**, Anti-6xHis immunoblots of input, soluble, and insoluble protein fractions from *E. coli* expressing GFP or *X. laevis* histones induced by 0.1 mM (**d**) and 10 mM (**e**) rhamnose. Each experiment was repeated at least twice independently with equivalent results. Units for molecular weight markers are noted for each figure: bp (base pair), KDa (kilodalton). Source data

On the basis of the structural predictions, we tested whether viral histone quadruplets form nucleosomes by selecting representatives from each repeat configuration, alongside two archaeal histones from *Methanothermus fervidus* (HmfA, HmfB), which we expressed in *E. coli*, a bacterium natively lacking histones. Green fluorescent protein (GFP) was included as an overexpression control. Immunoblotting revealed the production of each protein at comparable levels, except for the archaeal histones (Fig. 3a). However, differences in molecular weight and composition make blotting comparisons unreliable. Nonetheless, HQA and HQC histones were largely expressed and maintained as individual proteins, whereas HQB consistently fragmented, despite avoiding degradative conditions (Fig. 3a). Following expression, we investigated whether histone quadruplets form nucleosomal structures using micrococcal nuclease (MNase), an enzyme that degrades non-nucleosomal DNA^23^. As expected, *E. coli* containing empty vectors or expressing GFP exhibited no DNA protection following MNase digestion (Fig. 3b and Extended Data Fig. 6a). In contrast and consistent with previous results^24^, archaeal histones produced regular fragments with a primary size of ~70 bp, probably generated by tetramers, which increased successively by 30 bp, probably following dimer stacking (Fig. 3b and Extended Data Fig. 6a,b)^24,25^. Similar to archaeal histones, HQA and HQC quadruplets produced protected fragments but with an average primary size of 148 bp (141–156 bp), matching eukaryotic nucleosomes^26^. HQB provided variable protection, perhaps resulting from fragmentation. HQA and HQC quadruplets also generated higher-molecular-weight DNA fragments which increased on average by 90% (84%–97%) of the primary fragment size (Fig. 3b and Extended Data Fig. 6a,b). This increase differs from the digestion profile of eukaryotic chromatin, where higher-order fragments are produced from multiple nucleosomes and linker DNA (often between 20 and 80 bp). This suggests that histone quadruplets may stack or oligomerize without linker DNA, similar to archaeal histones^25^ and consistent with the absence of linker DNA between Marseillevirus nucleosomes^16^. Notably, although predicted HQA structures had a positively charged edge capable of DNA binding, similar to eukaryotic and archaeal nucleosomes, stacking was associated with a more neutral nucleosome face, as in HQA2, HQA5 and archaeal histones (Extended Data Fig. 5c). This contrasts with the positively charged faces of HQA4 and eukaryotic nucleosomes, suggesting that face charge repulsion could restrict stacking.

To characterize these putative oligomers, we crosslinked histones in vivo and assessed their molecular weights using immunoblotting (Fig. 3c). As predicted, archaeal histone HmfA and histone quadruplets HQA2, HQB2 and HQC2 displayed higher-order bands following crosslinking, with molecular weights approximately double or triple that of the monomers, consistent with oligomerization rather than non-specific binding. This implies that histone quadruplets form dimers and potentially higher-order structures (for example, HQA2). To corroborate these in vivo results, we tested HQA2 activity in vitro using electrophoretic mobility shift assays (EMSAs). HQA2 and HmfA were purified and incubated with a 147 bp nucleosome assembly sequence (Widom 601 DNA) (Fig. 3d–f)^27^. To improve comparability, both experiments were conducted under high salt conditions (2 M KCl), which was required for protein solubility. Mobility shifts indicated that HQA2 and HmfA bound DNA in vitro (Fig. 3e,f), and the two stepwise shifts observed at low and high HQA2 concentrations probably indicate dimer formation following monomer addition, similar to archaeal histones which also produced multiple shifts, albeit at a lower molecular weight (Fig. 3f). Normally, salt-gradient dialysis is required for in vitro nucleosome reconstitution; this implies that HQA2 could have a greater propensity for assembly relative to eukaryotic histones^28^. Although additional structural studies will be required, these data indicate that histone quadruplets can be expressed as repeats, assemble without native chromatin machinery, dimerize and bind DNA, forming eukaryotic-like nucleosomes that stack into archaeal-like oligomers, possibly through dimer or monomer addition (Fig. 3g).

Nucleosome assembly in eukaryotes typically requires chaperones (for example, NAP1, ASF1, CAF1 (ref. ^29^)), yet viral histone quadruplets formed in *E. coli* without eukaryotic proteins and assembled in vitro without salt-gradient dialysis. To understand histone quadruplet assembly, we tested the role of the amino acid linkers in nucleosome formation by generating two constructs containing the four core histones from the frog, *Xenopus laevis* (Fig. 3h). These histones were either expressed polycistronically in stoichiometric amounts using repeated ribosome binding sites^30^, or were conjoined using HQA1 linkers. Both constructs were expressed in *E. coli* and protein production was confirmed by immunoblotting (Fig. 3h). Micrococcal nuclease digestions revealed that both separated and fused *X. laevis* histones protected DNA. However, when separated, the DNA footprint was varied and smeared, similar to HQB (Fig. 3i). In contrast, fused *X. laevis* histones generated a defined singular band at 152 bp similar to canonical nucleosomes (Fig. 3i and Extended Data Fig. 6c). This fragment was present but less apparent when histones were separated. These experiments indicate that eukaryotic histones assemble into nucleosomes without native chaperones, but fusion facilitates assembly, perhaps by improving folding, stoichiometry and binding consistency. Protein misfolding in *E. coli* typically results in aggregation and incorporation into insoluble inclusion bodies^31^. Therefore, we inspected the solubility of these histones when expressed at varying levels by adjusting rhamnose inducer concentrations (Fig. 3j and Extended Data Fig. 6d,e). Although separated histones, alongside GFP, were consistently detected in the insoluble fraction, the fused histone was nearly absent. Moreover, GFP insolubility was dependent on expression level, unlike the separated histones which were consistently insoluble (Extended Data Fig. 6d,e). This suggests that histone insolubility is a result of ineffective assembly rather than overexpression, an effect mitigated by fusion.

### Viral histones impact genomic function and DNA compaction

**Fig. 4:**
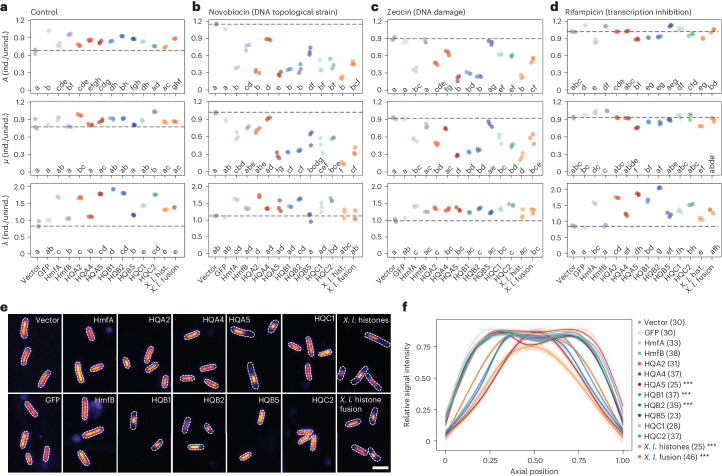
Histone quadruplets impact genomic function and nucleoid morphology in *Escherichia coli.* Growth characteristics inferred from OD_600_ measurements including maximum growth (*A*), maximum growth rate (*µ*) and lag phase (*λ*) in induced compared to uninduced strains. **a**–**d**, Growth parameters were inferred for three biological replicates under standard conditions (**a**) or with the addition of 150 µM novobiocin (**b**), 1 µM zeocin (**c**) or 1 µM rifampicin (**d**). Conditions were compared using pairwise Tukey honestly significant difference (HSD) tests. Significance groups are denoted using compact letter display (*P* < 0.01 after Bonferroni multiple test correction). See Extended Data Fig. 6 for full growth curves. **e**, Representative confocal fluorescent micrographs of *E. coli* cells after staining with Hoescht. Images are coloured using the Fire LUT (Lookup table) in ImageJ to show fluorescent intensity, and cell outlines are marked with dashed lines on the basis of brightfield microscopy. Scale bar, 2 µm. **f**, Hoescht staining signal intensity measured along the long axis of *E. coli* cells, normalized to the maximum intensity value and averaged across individuals (sample size is noted in parentheses). Shaded regions represent standard error. Only cells of consistent length (2–4 µm) were compared (ANOVA, *P* = 0.103, *F* = 1.535, d.f. = 13). Each experiment was repeated at least twice independently with equivalent results. ***P < 10^−20^, Kolmogorov–Smirnov test with Bonferroni correction, each strain compared to the empty vector control, *D* ≥ 0.15.

**Extended Data Fig. 7:**
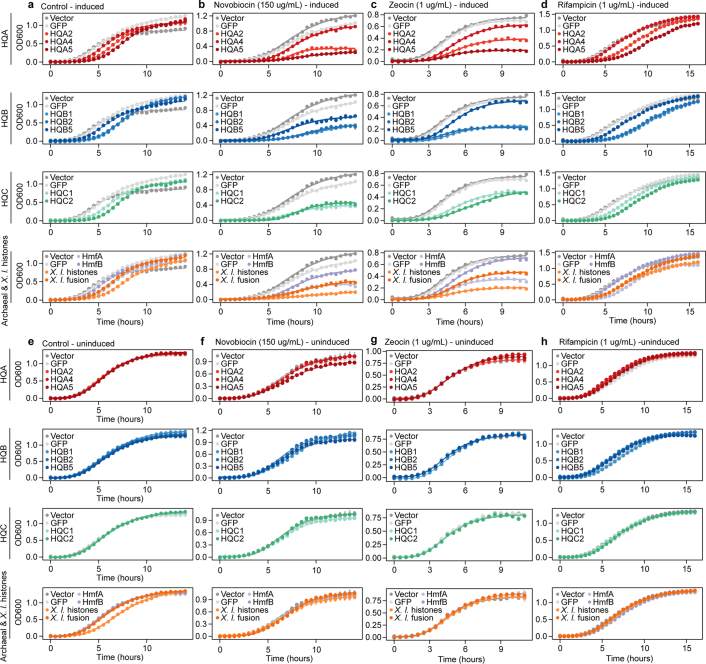
Complete *Escherichia coli* growth curves from which growth parameters were inferred. OD_600_-based growth curves for *E. coli* expressing different constructs when induced (**a-d**) or uninduced (**e-h**) under normal conditions (**a**, **e**) or in the presence of novobiocin (**b**, **f**), zeocin (**c**, **g**), or rifampicin (**d**, **h**). Data were fit to sigmoidal models. Points represent values averaged across three biological replicates and error bars represent the standard error of the mean. Note that the error bars are often very small and can be difficult to visualize. Each experiment was repeated at least three times independently with equivalent results.

Given the ability of histone quadruplets to assemble into nucleosome-like structures in *E. coli* and the known regulatory capacity of nucleosomes^2^, we assessed the phenotypic impact of histone expression in *E. coli*. Under standard conditions, histone production had a small or undetectable effect on maximum growth (*A*) and growth rate (*µ*) but increased lag phase (*λ*), suggesting a disruption to the transition out of stationary phase, rather than to the cell cycle (Fig. 4a and Extended Data Fig. 7). Histone expressing strains were also sensitive to antibiotics targeting genomic activities. Histone expression resulted in high sensitivity to novobiocin, a DNA gyrase inhibitor that causes excessive supercoiling, as well as zeocin, which induces double-stranded DNA breaks (Fig. 4b,c). Drug-challenged strains exhibited impaired growth but had normal lag phases relative to uninduced cultures. These effects were less pronounced in the presence of rifampicin, a transcription inhibitor, where growth dynamics mirrored the control (Fig. 4d). However, low concentrations of rifampicin were used to mediate transcriptional defects. Regardless, these synthetic lethal phenotypes indicate that histone quadruplets, similar to archaeal and *X. laevis* histones, affect genomic function probably by impacting DNA supercoiling, a canonical nucleosomal function^32^, and impairing DNA repair, perhaps by reducing genome accessibility.

Histone-dependent phenotypes also manifested as altered nucleoid morphology. In the presence of histone quadruplets HQA5, HQB1 and HQB2, and separated and fused *X. laevis* histones, *E. coli* nucleoids were condensed (Fig. 4e,f). This effect was not observed for other viral quadruplets or archaeal histones and was independent of cell size (*P* = 0.103) (Fig. 4f). Growth phenotypes, MNase digestion profiles and nucleoid condensation roughly correlated, although some quadruplets with well-defined digestion profiles (for example, HQA2, HQC1 and HQC2) did not alter nucleoid structure. These data indicate that amino acid sequence influences histone repeat function more than domain order and reveal another intermediate feature, as some histone quadruplets induce eukaryotic-like DNA condensation whereas others do not, similar to archaeal histones^24^. However, additional experimentation with other eukaryotic and archaeal histones could reveal more functional variability.

## Discussion

Here we examined the diversity of viral histones and further resolved histone phylogeny, corroborating previous analyses hinting at the ancient evolutionary history of histone repeats^11,17^. We demonstrate that histone quadruplets can self-assemble on a naïve genome where they form eukaryotic-like nucleosomes that stack into archaeal-like oligomers and affect genomic function. The formation of viral and eukaryotic nucleosomes in *E. coli* provides interesting experimental possibilities for synthetic chromatin biology.

Histone repeat evolution is complex, but these data permit a re-evaluation of three key origin hypotheses. First, viral histone repeats could have formed following the fusion of eukaryotic histones acquired by HGT after the last eukaryotic common ancestor (LECA). The rarity of histone repeats in eukaryotes would necessitate post-hoc fusion after viral acquisition, whereas the frequency of histone repeats in the marseilleviruses and iridoviruses is consistent with late histone acquisition. However, the NCV are thought to have diversified during eukaryogenesis^10^, which, together with the variability of viral genomes, metagenome incompleteness, variable host specificity and the lack of phylogenetic time calibration, makes the timing of viral histone acquisition unreliable. Regardless, this hypothesis is inconsistent with histone phylogenies placing histone repeats between archaea and eukaryotes, rather than within eukaryotes (Fig. 2a–e), and these topologies were independent of taxon sampling and long-branch attraction (Extended Data Fig. 3). The lack of conserved N-terminal residues in the viral repeats would also require significant and repeated divergence across each histone domain. Yet, the divergence of disordered terminal sequences is common during viral protein evolution^33^, making histone tails uninformative for reconstructing viral histone evolution.

A second hypothesis is that viral histone repeats represent the progenitors of eukaryotic histones, which relates to the viral karyogenesis model for the origin of the nucleus^34^. This could explain the emergence of the nucleosome before LECA, which is inferred under all models of the eukaryotic phylogeny. This hypothesis fits with the histone phylogenies, but the absence of additional chromatin proteins in NCV genomes either implies that viral chromatin proteins were transferred to a eukaryotic ancestor before being comprehensively lost in NCV lineages, or that eukaryotes evolved regulatory machinery after, rather than during, nucleosome evolution. Although histone linkage could permit nucleosome assembly, the absence of co-evolved regulatory proteins would have probably increased genomic sensitivity, complicating viral nucleosome acquisition. However, detrimental viral proteins may have driven chromatin evolution in dinoflagellates^35^.

**Fig. 5:**
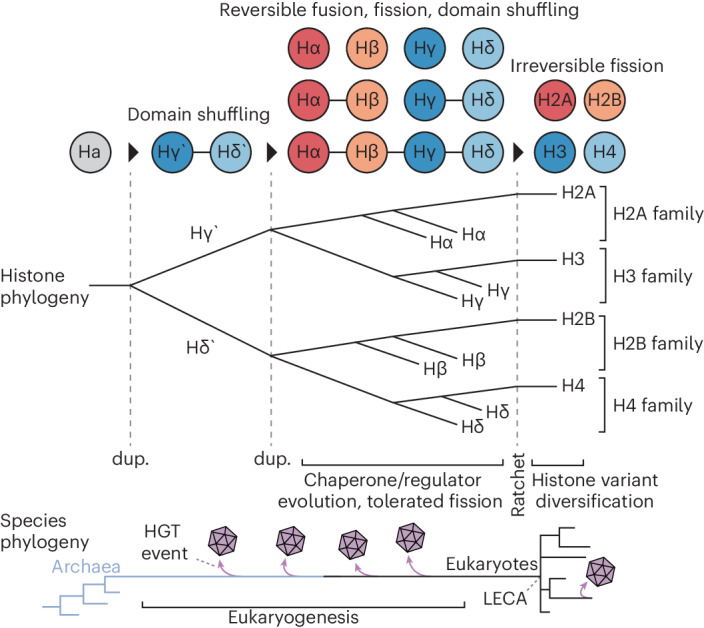
A hypothesis for the origin of the nucleosome. The top and bottom phylogenies illustrate hypothesized evolutionary histories for histones and species during eukaryogenesis, respectively. Key events in histone evolution are noted, including duplications (dup.) and an evolutionary ratchet, locking eukaryotic histones in their individualized states (for example, an evolved dependency on N-terminal tail modifications). HGT events are illustrated on the species phylogeny to demonstrate where viral histone repeats could have emerged from.

The third hypothesis is that histone repeats were acquired by NCV viruses from stem-eukaryotes during eukaryogenesis (Fig. 5)^11^. This is consistent with the phylogenies and the intermediate functional characteristics of the viral histone quadruplets. Likewise, the absence of key residues and additional chromatin proteins in the NCV could reflect an ancestral proto-eukaryotic state and host dependency, respectively. Importantly, reconstructing the exact evolutionary history of viral histone repeats may be impossible, but we suggest that this hypothesis is the most parsimonious as it agrees with our phylogenetic data, evokes the fewest atypical scenarios and is consistent with the evolution of other NCV genes which were probably acquired early in eukaryotic evolution^9,10^. If correct, these histones could represent molecular relics revealing snapshots of histone evolution during eukaryogenesis. These proteins and their contexts would have changed over time, particularly given elevated evolutionary rates in viruses^36^; however, the form and function of viral histone repeats can help dissect nucleosome evolution, and similar gene transfers involving other proteins could help resolve key aspects of the black box of eukaryogenesis.

Regardless of their evolution, viral histone repeats point to a hypothesis for the origin of the nucleosome (Fig. 5). By facilitating folding, histone linkers can resolve the paradox of whether assembly machinery or the nucleosome itself came first. We therefore hypothesize that the eukaryotic histones evolved through the repeated duplication of an archaeal homologue through repeat intermediates^4^. Based on our histone phylogeny, the progenitor initially duplicated into a primordial H3 (Hγ’) and H4 (Hδ’). H3 and H4 probably emerged first, given their ability to form tetramers and their initializing role in nucleosome formation^4^, and the infrequent co-occurrence of mixed pairs in viral genomes suggests functional incompatibility, indicating that a heterodimeric pair probably emerged first. The subsequent duplication of an Hγ’/ Hδ’ repeat would have formed the H2A/H3 and H2B/H4 families with pre-LECA representatives (Hα/Hγ, Hβ/Hδ, respectively^11^) (Fig. 5). We suggest that these existed as histone repeats, cooperatively assembling while chaperones and regulators evolved later to facilitate the process. Chaperones would have permitted fragmentation, potentiating histone fusion and fission. However, histone fusions have not been observed in eukaryotes. Therefore, we argue that an evolutionary ratchet occurred before LECA, such as a dependency on N-terminal tails or histone variants, which was incompatible with fusion, locking histones into separate proteins. Indeed, some archaeal histones are post-translationally modified and contain lysine-rich N-terminal tails, indicating that tail usage has been experimented with throughout histone evolution^37,38^. Ultimately, this scenario is consistent with genomic, phylogenetic and functional data and provides a parsimonious explanation for the origin of the nucleosome, a fundamental structure that impacted the emergence of core eukaryotic traits, from linear chromosomes to complex genome regulation and sex.

## Methods

### Histone identification and classification

To identify histone proteins in viral genomes, we created profile hidden Markov models (HMM) for each of the five eukaryotic histone families (including H1) and archaeal histones. To this end, we assembled a taxonomically balanced set of genome-predicted proteomes from UniProt^39^ (v.2021_03) by selecting the best proteome per genus from eukaryotes (*n* = 114) and Archaea (*n* = 230) on the basis of BUSCO (Benchmarking Universal Single Copy Orthologues) presence^40^. For opisthokonts (Metazoa and Fungi) and streptophytes, stricter criteria were used by selecting the best proteome per phylum (*n* = 24) and family (*n* = 11), respectively. The archaeal proteomes were also supplemented with additional non-reference Asgard proteomes from UniProt (*n* = 14) and the resulting proteomes from all species were separately clustered at 95% identity using CD-HIT (v.4.8.1)^41^.

Using histone orthologues from *Homo sapiens* (H1: P07305, H2A: Q96QV6, H2B: P682807, H3: P68431, H4: P62805) and *Methanothermus fervidis* (P48781) as separate queries, we searched the proteomes using Diamond BLASTp v.2.0.9 (ultra-sensitive mode, *E* < 10^−5^)^42,43^. The BLAST hits were extracted and aligned using the structurally informed MAFFT-DASH v.7.490 multiple sequence aligner with the L-INS-i algorithm, before being trimmed to a gap threshold of 20% using trimAl (v.1.4.rev15)^44,45^. Phylogenies were generated from the trimmed alignments using IQ-Tree v.2.1.2, and LG (Le-Gascuel) substitution models were selected using ModelFinder^46^. The trees were inspected using FigTree v.1.4 (http://tree.bio.ed.ac.uk/software/figtree/), and correct orthologues were identified and extracted on the basis of phylogenetic topologies and sequence annotation using SWISS-PROT^47^. The identified orthologues were then re-aligned using MAFFT-DASH and used to generate HMMs with HMMER (v.3.1b2)^48^. The HMMs were used to search the proteomes twice iteratively using HMMER (*E* < 10^−5^) and between searches, the hits were extracted, aligned with the previously identified homologues, phylogenetically curated and incorporated into improved HMMs representing each of the histone families and encompassing diversity from across eukaryotes and Archaea.

Each of the histone HMMs was subsequently used to search a database of proteins predicted from diverse Nucleocytoviricota genomes and assembled metagenomes^7^. To filter out incomplete or fragmented sequences, proteins were re-predicted from viral genomes using Prodigal v.2.6.3 and annotated as complete given the presence of a start and stop codon^49^. The resulting full-length viral histone homologues included histone repeat proteins. Therefore, to classify the histone domains found within individual proteins, the sequences were annotated with the histone HMMs using HMMER. When multiple HMMs overlapped by more than 50%, the domain with the better conditional *E*-value was assigned to the region, and domains mapping with less than 25% HMM coverage were excluded. Annotated histone domains were then extracted and concatenated with reference sequences from each of the eukaryotic and archaeal histone families before sequence alignment and phylogenetic analysis, as described above. Histone domains were annotated phylogenetically as belonging to the archaeal, H2A, H2B, H3 or H4 families. If a histone domain could not be assigned due to an intermediate branching position, it was denoted Hx (*n* = 2). The final histone types were classified on the basis of histone domain order and composition and, to avoid prediction artefacts, only histone types observed in more than one separate genome were analysed further.

To corroborate the genome predictions, we also searched for viral histone homologues in ocean metatranscriptomes produced as part of the Tara Oceans Project^18^. To reduce database size, unigenes annotated with a histone-fold domain based on Pfam (PF00125) were extracted. Transcripts were translated using TransDecoder v.5.5.0 (https://github.com/TransDecoder/TransDecoder), and the predicted proteins were searched and annotated as above using the histone HMMs.

### Reconstructing chromatin processing machinery in the Nucleocytoviricota

To understand co-occurrence between histone proteins and chromatin processing machinery in the Nucleocytoviricota, we searched Nucleocytoviricota genomes and metagenomes for a suite of previously curated chromatin-associated proteins^50^. These proteins included post-translational modifiers, chaperones, readers, remodellers and other proteins involved in complex formation from diverse eukaryotes. Previously identified orthologues were downloaded, aligned using the L-INS-i algorithm in MAFFT v.7.490, and HMMs were generated from the resulting alignments. Nucleocytoviricota proteomes were then searched using these HMMs with HMMER (*E* < 10^−5^), and if a single protein was identified by multiple HMMs, it was assigned to the HMM that produced the lower *E*-value. The resulting hits were visualized using a heat map, and hierarchical clustering was conducted using a correlation distance metric and the ward D2 clustering method implemented in Pvclust in R (v.4.2.0)^51^.

### Phylogenetics and topology testing

Phylogenies were generated for individual histone families and the entire histone superfamily using IQ-Tree. Individual histone domains were extracted from repeat proteins as described above and aligned with individual eukaryotic and archaeal histones using MAFFT-DASH v.7.490 and the L-INS-i algorithm. The resulting alignments were trimmed with a gap threshold of 20% and sequences with less than 50% of sites present were removed. Fast-evolving eukaryotic taxa including parasites and amoebozoans were excluded from the analyses due to phylogenetic ambiguity (for example, *Trichomonas, Naegleria, Dictyostelium*). For each phylogeny, substitution models were selected using ModelFinder and statistical support was generated using Shimodaira–Hasegawa approximate likelihood ratio tests (SH-aLRT, *n* = 1,000)^52^. Given the short alignments (~100 amino acids), we used a reduced perturbation strength (-pers 0.2), an increased threshold for unsuccessful tree search iterations (-nstop 200) and ran each tree search 100 times independently to avoid local likelihood maxima, selecting the best tree from each set of replicates. Note that phylogenies calculated using empirical profile mixture models were ran only once due to computational limitations. To account for phylogenetic artefacts including long-branch attraction and compositional bias, phylogenies were re-run using alternative substitution models, 4-state Dayhoff recoded alignments and alignments lacking compositionally biased sequences based on composition *χ*^2^ tests (*P* < 0.05) performed in IQ-Tree^53^. For topology testing, topological constraints were applied and constrained trees were calculated as above and compared to the best unconstrained tree using approximately unbiased (AU) tests^54^. For testing the monophyly of proteins with different domain configurations, only viral sequences branching outside of the eukaryotic clade in the best tree were constrained as monophyletic. Phylogenies were visualized in IToL (v.6)^55^.

### Sequence and structural analysis

Histone repeat sequences were analysed computationally to understand functional, structural and evolutionary characteristics. Predicted proteolytic cleavage sites were assessed using the PeptideCutter tool in ExPasy, whereas protein tertiary structure was calculated using AlphaFold v.2.3.2 implemented through ColabFold (v.1.5.5)^22,56,57^. For the AlphaFold predictions, structures were predicted using the AlphaFold 2-multimer-v2 model, given a dimeric configuration with multiple sequence alignments generated using homologues from the MMSeqs2 database^58^. Protein structure visualization and root mean square deviation (RMSD) calculations were done using Pymol v.2.5.0 (https://github.com/schrodinger/pymol-open-source). DNA was added to the models from an *X. laevis* nucleosome structure (PDB: 6ESF), following structural alignment in Pymol. Protein electrostatic predictions were made using the APBS v.3.4.1 webserver (https://server.poissonboltzmann.org/)^59^. To investigate eukaryotic sequence conservation, multiple sequence alignments and phylogenies for each histone family (excluding viral sequences) were analysed using ConSurf (v.1.0)^20^. Conservation comparisons between viral and eukaryotic sequences were done using TwinCons v.1.0 utilizing the LG substitution model and Voronoi clustering^19^. Shannon entropy was calculated using Bio3D v.2.4–4^60^ and multiple sequence alignments were visualized in AliView (v.1.28)^61^.

### Histone expression and immunoblotting

Archaeal, viral and eukaryotic histone genes with C-terminal 6xHis tags were expressed in *E. coli* strain K12 from a rhamnose-inducible promoter (pRha) in a pD861 vector after being codon optimized and synthesized by Synbio Technologies. *Xenopus laevis* histones (P06897, P02281, P02302, P62799) were either expressed polycistronically with each open reading frame separated by a ribosome binding site, or were joined with three linkers from histone quadruplet HQA1 (AEAEDVK, TELGELINKQLFNDDRKRKLATARRNRRKAEDTGDADGATTSG, QPARLNT)^30^. Plasmids were transformed into *E. coli* by heat shock and were maintained using kanamycin selection (50 µg ml^−1^). To induce histone expression, cells were grown overnight in LB (lysogeny broth) at 37 °C before being diluted 1:100 into fresh LB and grown at 30 °C to an optical density at 600 nm (OD_600_) of 0.6. before induction. Cultures were cold shocked in an ice water bath for 30 min while shaking at 100 r.p.m. to improve protein solubility after induction. The inducer, l-rhamnose monohydrate, was then added at 10 mM (unless otherwise noted) and cultures were induced for 18 h at 20 °C, shaking at 180 r.p.m.

Immunoblots were used to confirm protein expression and assess histone structural properties. Total protein extracts were collected by centrifuging 150 µl of induced culture at 15,000 × *g* for 2 min, resuspending the cell pellet to an OD_600_ of 1.0 in SDS loading buffer (50 mM Tris pH 6.8, 2% SDS, 10% glycerol, 100 mM β-mercaptoethanol) and incubating the extracts at 37 °C for 10 min. Protein extracts were loaded into 4–20% Tris-glycine SDS–PAGE gels (BioRad) and ran at 120 V for 30 min in Tris-glycine-SDS buffer (2.5 mM Tris, 19.2 mM glycine, 0.01% SDS, pH 8.3). Polyacrylamide gels were equilibrated for 15 min in transfer buffer (2.5 mM Tris, 19.2 mM glycine, 20% methanol, pH 8.3) at room temperature and then transferred onto 0.2-µm-pore-size nitrocellulose membranes at 30 V for 70 min at 4 °C. Total transferred protein was assessed using Pierce Reversible Protein stain (Thermo Fischer), and membranes were blocked for 2 h at room temperature with 0.2% alkali soluble casein in PBST (10 mM Na_2_HPO_4_, 1.8 mM KH_2_PO_4_, 150 mM NaCl, 0.1% Tween-20, pH 7.5). Blocked membranes were incubated for 1 h at room temperature in 0.2 µg ml^−1^ anti-6x His-tag (ab9108) in PBS-Tween after conjugating the antibody to horseradish peroxidase (HRP) using an HRP conjugation kit (ab102890). Finally, membranes were washed in PBST and chemiluminescence was visualized using Pierce ECL (enhanced chemiluminescence) substrate and an Invitrogen iBright CL1500 imaging system.

### Micrococcal nuclease digestions

To assess nucleosome formation, *E. coli* cultures were induced, fixed, lysed and digested with micrococcal nuclease, an enzyme that preferentially digests non-nucleosomal DNA, following previously published methods^24^. Induced cultures (10 ml) were prepared as described above, before being fixed in 1% formaldehyde for 15 min while shaking at 180 r.p.m. Fixation was quenched with 125 mM glycine for 5 min and the resulting cells were pelleted, washed in cold PBS and stored at −80 °C. Thawed cell pellets were then resuspended and digested for 1 h on ice with freshly made lysozyme buffer (120 mM Tris pH 8, 50 mM EDTA, 4 mg ml^−1^ lysozyme (Thermo Fisher, 89833)). Protoplasts were pelleted at 15,000 × *g* for 3 min, resuspended in 500 µl of cold lysis buffer (10 mM NaCl, 10 mM Tris pH 8, 3 mM MgCl_2_, 0.5% NP-40, 0.15 mM spermine, 0.5 mM spermidine, Roche EDTA-free protease inhibitor) and incubated on ice for 20 min. Lysates were pelleted at 15,000 × *g* for 10 min and the resulting pellets were gently washed in −CA buffer (15 mM NaCl, 10 mM Tris pH 7.4, 60 mM KCl, 0.15 mM spermine, 0.5 mM spermidine, EDTA-free protease inhibitor) without resuspension. Lysates were then centrifuged at 15,000 × *g* for 5 min and resuspended in 500 µl of cold +CA buffer (15 mM NaCl, 10 mM Tris pH 7.4, 5 mM CaCl_2_, 60 mM KCl, 0.15 mM spermine, 0.5 mM spermidine, EDTA-free protease inhibitor). Before digestion, samples were equilibrated to 37 °C before micrococcal nuclease (Thermo Fisher, 88216) was added at 250 U ml^−1^. Lysates were then briefly vortexed and incubated at 37 °C, with samples being taken at designated time points. To stop the reaction, 0.25 volumes of STOP solution (200 mM EDTA pH 8, 200 mM EGTA pH 8) were added after sample collection. Lastly, digested samples were treated with 400 µg ml^−1^ RNaseA (Thermo Fisher, EN0531) for 30 min at 37 °C, followed by 1% SDS and 800 µg ml^−1^ proteinase K (Thermo Fisher EO491) for 16 h at 65 °C. DNA was purified using a Qiagen PCR purification kit, and samples were analysed using 2.5% agarose TBE gels (90 mM Tris, 90 mM boric acid, 2 mM EDTA pH 8) which were electrophoresed at 150 V for 30 min. DNA fragment sizes were quantified using an Agilent 2100 Bioanalyzer with a high-sensitivity DNA kit.

### Glutaraldehyde crosslinking

Induced cultures (10 ml) were prepared, and the cells pelleted and washed with cold PBS before being frozen at −80 °C. Thawed cell pellets were resuspended and digested in 1 ml of fresh lysozyme buffer on ice for 1 h to generate protoplasts. The cells were then resuspended in 500 µl of cold lysis buffer (10 mM NaCl, 10 mM HEPES pH 7.5, 3 mM MgCl_2_, 0.5% NP-40, 0.15 mM spermine, 0.5 mM spermidine, EDTA-free protease inhibitor) using a 22-gauge syringe and left to incubate on ice for 20 min. Lysates were centrifuged at 15,000 × *g* for 10 min and the supernatant was removed before the pellet was again resuspended in 500 µl cold HEPES buffer (10 mM NaCl, 10 mM HEPES pH 7.5, 30 mM KCl, 3 mM MgCl_2_, 0.15 mM spermine, 0.5 mM spermidine, EDTA-free protease inhibitor) using a 22-gauge syringe. Samples were incubated at 20 °C for 5 min and split into 95 µl aliquots to which either water or glutaraldehyde was added to a final concentration of 0.025%. The samples were fixed at room temperature for 5 min before being quenched with 100 mM Tris pH 7.6. Fixed samples were then treated with 400 µg ml^−1^ RNaseA for 30 min at 37 °C and 2.5 units of DNAseI (Thermo Fisher, EN0525) for 1 h at 37 °C. SDS loading buffer was added to the samples which were denatured at 95 °C for 10 min before being analysed by SDS–PAGE and immunoblotting, as described above, but with prolonged electrophoresis and transfer times of 1 h.

### Protein purification and electrophoretic mobility shift assays

Cultures (900 ml) were induced, pelleted in 150 ml increments, washed with PBS and stored at −80 °C, as described above. Thawed cell pellets were then resuspended in 1 ml of cold lysozyme buffer and incubated on ice for 1 h. The resulting protoplasts were pelleted at 15,000 × *g* for 3 min and washed with 1 ml of cold PBS without resuspension. The pellet was then resubmerged in 1 ml of cold lysis buffer (50 mM HEPES pH 7.5, 2 M NaCl, 25 mM imidazole, Roche EDTA-free protease inhibitor) and sonicated five times using a Soniprep 150 sonicator (MSE) at 50% power (10 amplitude microns) for 30 s with 3 min ice incubations between cycles. Lysates were then pelleted at 16,000 × *g* for 20 min at 4 °C. The soluble supernatants were then pooled and the volume was topped up to 12 ml with equilibration buffer (1 M NaCl, 25 mM imidazole, 10 mM Na_2_HPO_4_, 1.8 mM KH_2_PO_4_). Protein purification was then accomplished using 3 ml HisPur Ni-NTA spin columns (Thermo Fisher), following supplier instructions. Lysates were added to the Ni-NTA resin and incubated for 30 min while rotating at 15 r.p.m. at 4 °C. The columns were subsequently washed five times with wash buffer (1 M NaCl, 50 mM imidazole, 10 mM Na_2_HPO_4_, 1.8 mM KH_2_PO_4_) for 5 min while rotating at 4 °C. Finally, proteins were eluted three times with elution buffer (50 mM HEPES pH 7.5, 2 M KCl, 250 mM imidazole, Roche EDTA-free protease inhibitor) following a 10 min incubation while rotating at 4 °C. The resulting elutions were dialysed overnight to remove the imidazole using 3.5 kDa Slide-A-Lyzer dialysis cups (Thermo Fisher), and proteins were quantified using a Qubit fluorometer (Thermo Fisher). Purification and molecular weights were assessed using 4–20% Tris-glycine SDS–PAGE gels (ran at 150 V for 30 min) that stained overnight with InstantBlue Coomassie protein stain (Abcam).

To conduct EMSAs, each protein was incubated at varying concentrations (from 1–10 µM) with 1 µM 147 bp Widom 601 DNA fragments in binding buffer (50 mM HEPES pH 7.5, 2 M KCl, 1 mM EDTA pH 8, 20% glycerol). Widom 601 DNA fragments were amplified from a pGEM-3z/601 plasmid (a gift from Jonathan Widom, Addgene plasmid 26656; http://n2t.net/addgene:26656; RID:Addgene_26656) by polymerase chain reaction using forward (5′-CTGGAGAATCCCGGTGCCG-3′) and reverse (5′-ACAGGATGTATATATCTGACACG-3′) primers and Econo*Taq* mastermix (Biosearch Tech) following 35 cycles with an annealing temperature of 55 °C and an extension time of 30 s. The resulting amplicons were purified by ethanol precipitation and quantified using a Nanodrop spectrophotometer (Thermo Fisher). Mobility shifts were assessed by electrophoresis using 5% TBE polyacrylamide gels (BioRad). Samples were run at 150 V for 40 min at room temperature in 0.5x TBE (44.5 mM Tris, 44.5 mM boric acid, 1 mM EDTA pH 8) and the resulting gels were stained with SYBR GOLD DNA stain (Thermo Fisher) in 0.5x TBE for 15 min before imaging using an iBright CL1500.

### Histone solubility assays

To assess the influence of histone linkers on protein solubility, *E. coli* strains (10 ml cultures) expressing fused or separated *Xenopus* histones were induced and frozen as described. To lyse the cells, cell pellets were resuspended and incubated in lysozyme buffer on ice for 1 h. Protoplasts were then pelleted at 15,000 × *g* for 3 min at 4 °C and resuspended in 500 µl of cold HEPES lysis buffer. To ensure efficient lysis, the resuspended cells were then sonicated three times using a Soniprep 150 sonicator at 50% power (10 amplitude microns) for 10 s with a 1 min ice incubation between cycles. NP-40 was then added at 0.5% and the lysates were mixed well and incubated on ice for 20 min. An input sample was then taken and the remaining lysates were centrifuged at 16,000 × *g* for 20 min at 4 °C. The soluble supernatant was isolated and the remaining insoluble pellet was washed once with PBS. The insoluble pellet was then resuspended in lysis buffer and sampled. Protein samples were mixed with SDS loading buffer, denatured at 95 °C for 10 min and analysed by SDS–PAGE and immunoblotting.

### Growth curves and phenotyping

To investigate the phenotypic impacts of histone expression in *E. coli*, strains were inoculated and grown overnight in 5 ml of LB medium with kanamycin (LB-Kan). The next day, cultures were diluted 1:100 into 5 ml of fresh LB-Kan and grown to an OD_600_ of 0.6 at 37 °C. The cultures were then cold shocked in an ice water bath for 30 min while shaking, before rhamnose monohydrate was added at a concentration of 10 mM. Induced cultures were then grown at 30 °C for 4 h, shaking at 225 r.p.m., before being inoculated to an initial OD_600_ of 0.025 in a 96-well plate containing 200 µl of LB-Kan supplemented with either novobiocin (150 µg ml^−1^), rifampicin (1 µg ml^−1^) or zeocin (1 µg ml^−1^). Growth was monitored at 30 °C with 400 r.p.m. double orbital shaking using a FLUOstar Omega (BMG Labtech) plate reader, with measurements conducted every 30 min at 600 nm. The resulting data were collected and the OD_600_ of uninoculated media was subtracted from each measurement. Sigmoidal growth curves were then fit to individual biological replicates using a nonlinear least square fitting function (maximum iterations = 10,000, tolerance = 1 × 10^−5^) based on the formula:

$$y=A{e}^{-{\rm{e}}^{{\rm{\mu }}e/A(\lambda -{\rm{time}}+1)}},$$

where *µ*, *A* and *λ* represent the maximum growth rate defined as the maximum slope, maximum growth interpreted as the curve maximum, and the lag-phase time determined as the time of the maximum slope, respectively. All analyses were conducted in R v.4.2.0.

### Nucleoid imaging

Induced *E. coli* strains were prepared as described above, fixed with 0.2% glutaraldehyde and incubated with 15 µg ml^−1^ Hoescht 33342 for 30 min in the dark at room temperature. Stained cells were then applied to a slide and mixed with an equal volume of 50% CitiFluor AF2 antifade solution in PBS. Slides were sealed and imaged using a Zeiss LSM-780 inverted high-resolution laser scanning confocal microscope with a Ph3 ×100 oil objective. Exposures were kept constant during experiments, and images were collected using Zeiss ZEN Black Software (ZEN Digital Imaging for Light Microscopy) and analysed with ImageJ v.1.54 (https://imagej.net). Nucleoid density was measured in ImageJ by measuring the fluorescent signal intensity along a transect between cell ends, determined using brightfield microscopy. Signal intensity was made relative to the maximum measured value and averaged across measured cells. Only cells that were clearly non-dividing and lying flat in the focal plane were measured. Likewise, to control for variation in cell size, only cells between 2–4 µm were included in the analysis. Nucleoid distributions were compared using Kolmogorov–Smirnov tests conducted in R.

### Reporting summary

Further information on research design is available in the Nature Portfolio Reporting Summary linked to this article.

### Supplementary information

Reporting Summary

### Source data

Source Data Fig. 3 Unprocessed immunoblots and gels for Fig. 3.

Source Data Extended Data Fig. 6 Unprocessed immunoblots and gels for Extended Data Fig. 6.

Source Data Extended Data Fig. 1 Eukaryotic and archaeal UniProt proteome accessions.

## Figures and Tables

**Extended Data Table 1:**
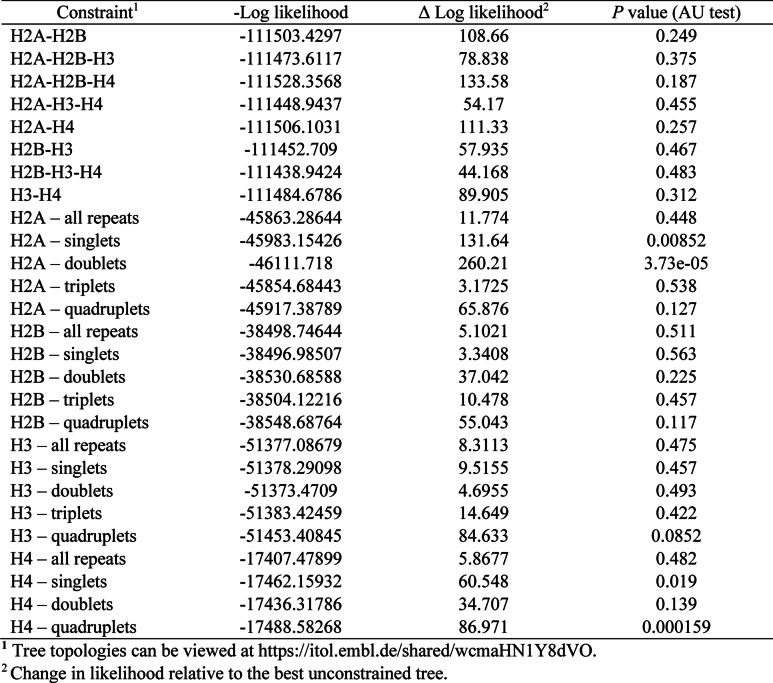
Constrained histone phylogenies and topology testing

## Data Availability

All datasets used in the study are available from figshare (https://figshare.com/s/40c5ee5552097be43c6b)^62^. The identifiers of all eukaryotic and archaeal proteomes used in this study are listed in the provided Source data. Viral proteomes were downloaded from https://figshare.com/s/14788165283d65466732 (ref. ^63^). Requests and correspondence can be addressed to N.A.T.I. (nicholas.irwin@gmi.oeaw.ac.at). Source data are provided with this paper.
