# Supplementary material for: Self-assembling viral histones are evolutionary intermediates between archaeal and eukaryotic nucleosomes
Source: Nat Microbiol. 2024 May 28;9(7):1713–24. doi: 10.1038/s41564-024-01707-9 (PMC11222145; doi:10.1038/s41564-024-01707-9)
Supplement: Supplementary file 1 — Reporting Summary [file 41564_2024_1707_MOESM1_ESM.pdf]

nature portfolio

Corresponding author(s): Nicholas Irwin

Last updated by author(s): Aug 4, 2023

Reporting Summary

Nature Portfolio wishes to improve the reproducibility of the work that we publish. This form provides structure for consistency and transparency in reporting. For further information on Nature Portfolio policies, see our Editorial Policies and the Editorial Policy Checklist.

Statistics

For all statistical analyses, confirm that the following items are present in the figure legend, table legend, main text, or Methods section.

n/a	Confirmed	
☐	☒	The exact sample size (*n*) for each experimental group/condition, given as a discrete number and unit of measurement
☒	☐	A statement on whether measurements were taken from distinct samples or whether the same sample was measured repeatedly
☐	☒	The statistical test(s) used AND whether they are one- or two-sided<br>*Only common tests should be described solely by name; describe more complex techniques in the Methods section.*
☒	☐	A description of all covariates tested
☐	☒	A description of any assumptions or corrections, such as tests of normality and adjustment for multiple comparisons
☐	☒	A full description of the statistical parameters including central tendency (e.g. means) or other basic estimates (e.g. regression coefficient) AND variation (e.g. standard deviation) or associated estimates of uncertainty (e.g. confidence intervals)
☐	☒	For null hypothesis testing, the test statistic (e.g. *F*, *t*, *r*) with confidence intervals, effect sizes, degrees of freedom and *P* value noted<br>*Give P values as exact values whenever suitable.*
☒	☐	For Bayesian analysis, information on the choice of priors and Markov chain Monte Carlo settings
☒	☐	For hierarchical and complex designs, identification of the appropriate level for tests and full reporting of outcomes
☒	☐	Estimates of effect sizes (e.g. Cohen's *d*, Pearson's *r*), indicating how they were calculated

Our web collection on statistics for biologists contains articles on many of the points above.

Software and code

Policy information about availability of computer code

Data collection	Zeiss ZEN Black 2.3
Data analysis	The following software was used for data analysis: CD-HIT v4.8.1, Diamond BLASTp v2.0.9, MAFFT v7.490, Trimal v1.4.rev15, IQ-Tree v2.1.2, FigTree v1.4, HMMER v3.1b2, Prodigal v2.6.3, TransDecoder v5.5.0, R v.4.2.0, ModelFinder, IToL v6, AlphaFold v2.3.2, Expasy PeptideCuttter, ColabFold v1.5.5, Pymol v2.5.0 OpenSource, Consurf v1.0, TwinCons v1.0, ABPS v3.4.1, Bio3D v2.4-4, Aliview v1.28, ImageJ v1.54

For manuscripts utilizing custom algorithms or software that are central to the research but not yet described in published literature, software must be made available to editors and reviewers. We strongly encourage code deposition in a community repository (e.g. GitHub). See the Nature Portfolio guidelines for submitting code & software for further information.

Data

Policy information about availability of data

All manuscripts must include a data availability statement. This statement should provide the following information, where applicable:

- Accession codes, unique identifiers, or web links for publicly available datasets
- A description of any restrictions on data availability
- For clinical datasets or third party data, please ensure that the statement adheres to our policy

All datasets used in the study are available from FigShare (https://figshare.com/s/40c5ee5552097be43c6b). The identifiers of all eukaryotic and archaeal proteomes

used in this study are listed in the provided Source Data. Viral proteomes were downloaded from https://figshare.com/s/14788165283d65466732. Requests and correspondence can be addressed to N.A.T.I. (nicholas.irwin@gmi.oeaw.ac.at).

Research involving human participants, their data, or biological material

Policy information about studies with human participants or human data. See also policy information about sex, gender (identity/presentation), and sexual orientation and race, ethnicity and racism.

Reporting on sex and gender	NA
Reporting on race, ethnicity, or other socially relevant groupings	NA
Population characteristics	NA
Recruitment	NA
Ethics oversight	NA

Note that full information on the approval of the study protocol must also be provided in the manuscript.

Field-specific reporting

Please select the one below that is the best fit for your research. If you are not sure, read the appropriate sections before making your selection.

☒ Life sciences ☐ Behavioural & social sciences ☐ Ecological, evolutionary & environmental sciences

For a reference copy of the document with all sections, see nature.com/documents/nr-reporting-summary-flat.pdf

Life sciences study design

All studies must disclose on these points even when the disclosure is negative.

Sample size	No calculations were used to predetermine sample size. Sample sizes were maximized based on experimental limitations.
Data exclusions	No data were excluded.
Replication	All experiments were repeated a minimum of two times. Consistent results were obtained during replication.
Randomization	Randomization was not relevant in our study because there was not a risk of selection bias given the experiments we were conducting.
Blinding	Blinding was not relevant in our study because there was little risk of observation bias.

Reporting for specific materials, systems and methods

We require information from authors about some types of materials, experimental systems and methods used in many studies. Here, indicate whether each material, system or method listed is relevant to your study. If you are not sure if a list item applies to your research, read the appropriate section before selecting a response.

Materials & experimental systems

n/a	Involved in the study	
☐	☒	Antibodies
☒	☐	Eukaryotic cell lines
☒	☐	Palaeontology and archaeology
☒	☐	Animals and other organisms
☒	☐	Clinical data
☒	☐	Dual use research of concern
☒	☐	Plants

Methods

n/a	Involved in the study	
☒	☐	ChIP-seq
☒	☐	Flow cytometry
☒	☐	MRI-based neuroimaging

Antibodies

Antibodies used	anti 6xHis tag (AbCam, ab9108) - 0.2 µg/mL

Validation	The antibody was verified by AbCam by ELISA and was deemed to be suitable for immunoblot (Western blot). More information can be found at: https://www.abcam.com/products/primary-antibodies/6x-his-tag-antibody-ab9108.html
