# Supplementary material for: Self-assembling viral histones are evolutionary intermediates between archaeal and eukaryotic nucleosomes
Source: Nat Microbiol. 2024 May 28;9(7):1713–24. doi: 10.1038/s41564-024-01707-9 (PMC11222145; doi:10.1038/s41564-024-01707-9)

Uncropped and unedited gel and blot images for Figure 3.

Figure 3a.
chemiluminescence

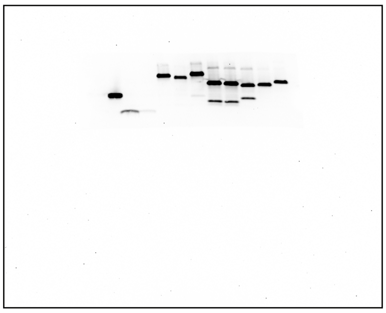

chemiluminescence-membrane overlay

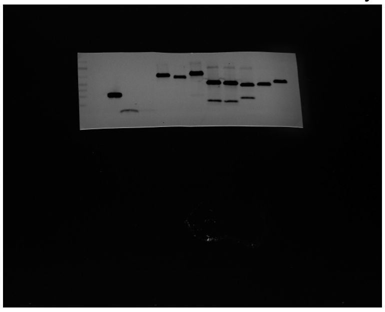

total protein

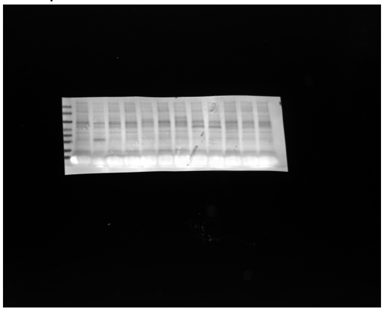

Figure 3b.
SYBR-Safe fluorescence

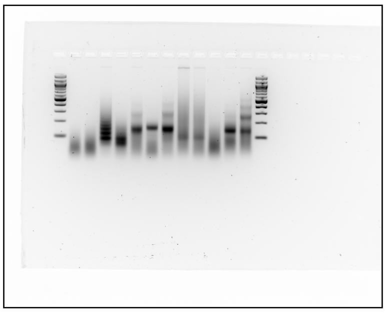

Figure 3d.
Coomassie

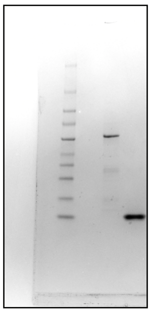

Figure 3e.
SYBR-Gold fluorescence

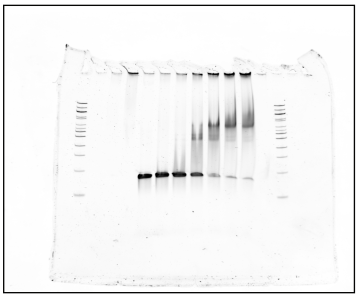

Figure 3f.
SYBR-Gold fluorescence

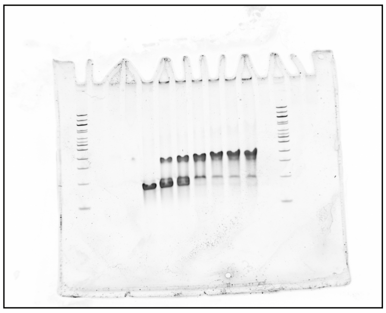

Figure 3c.
chemiluminescence

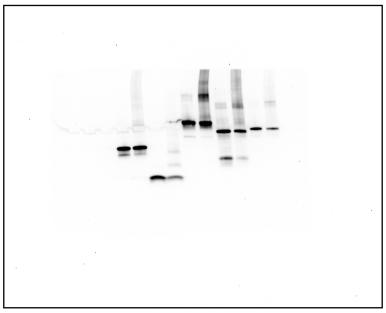

chemiluminescence-membrane overlay

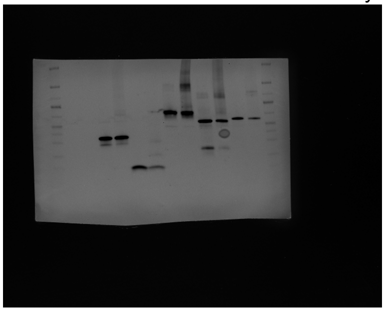

total protein

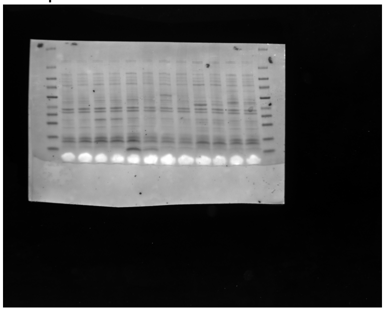

Figure 3h.
chemiluminescence

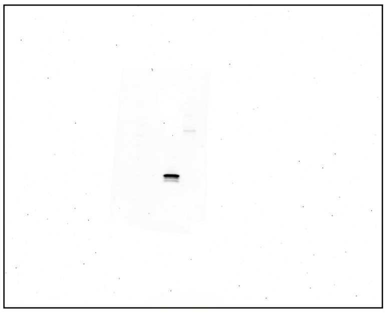

chemiluminescence-membrane overlay

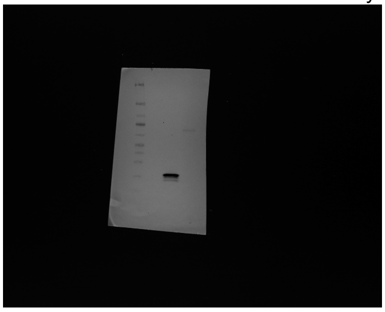

total protein*

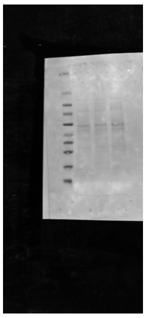

*Note that the membrane was cut after protein staining

Figure 3i.
SYBR-Safe fluorescence

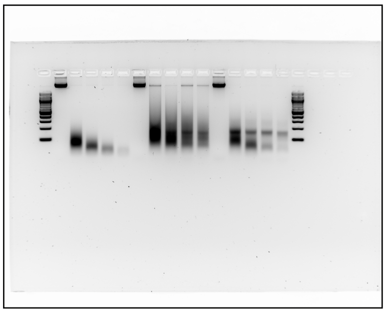

Figure 3j.
chemiluminescence

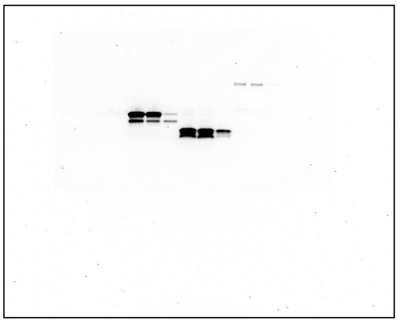

chemiluminescence-membrane overlay

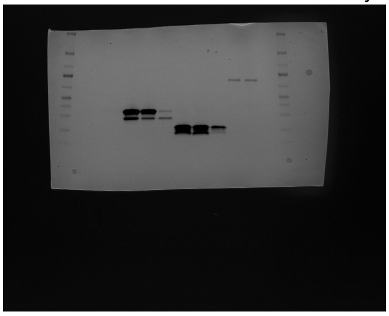

total protein

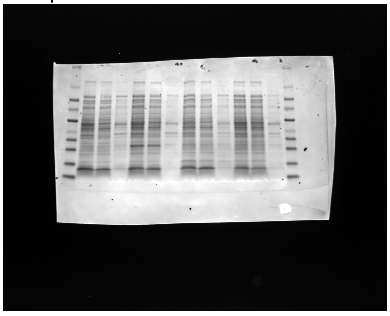

Supplement: Supplementary file 2 — Unprocessed immunoblots and gels for Fig. 3. [file 41564_2024_1707_MOESM2_ESM.pdf]
