# Supplementary material for: Self-assembling viral histones are evolutionary intermediates between archaeal and eukaryotic nucleosomes
Source: Nat Microbiol. 2024 May 28;9(7):1713–24. doi: 10.1038/s41564-024-01707-9 (PMC11222145; doi:10.1038/s41564-024-01707-9)

Uncropped and unedited gel and blot images for Extended Data Figure 6

Extended Data Fig. 6a
SYBR-Safe fluorescence

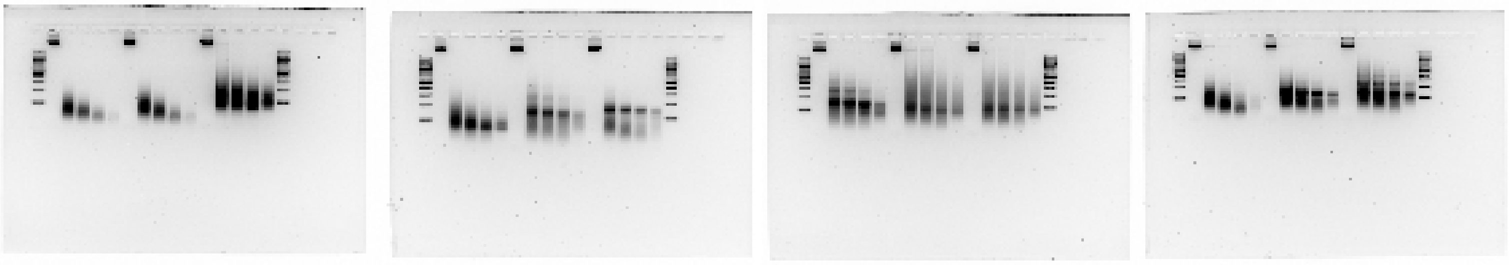

Extended Data Fig. 6d
chemiluminescence

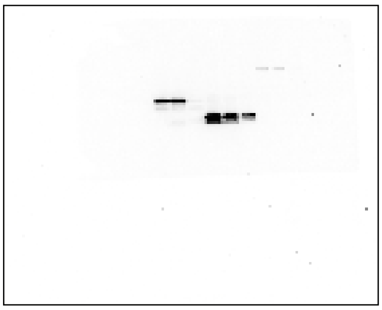

chemiluminescence-membrane overlay

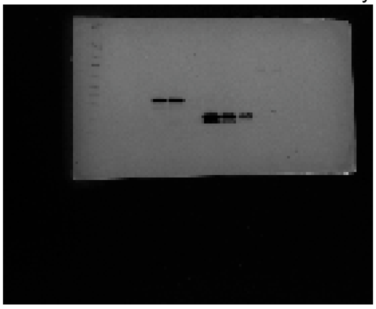

total protein

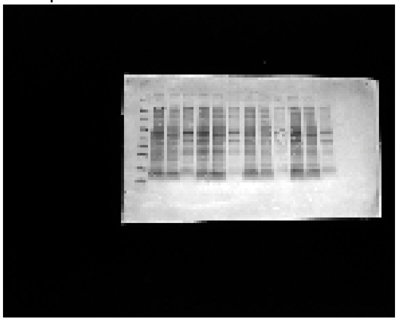

Extended Data Fig. 6e
chemiluminescence

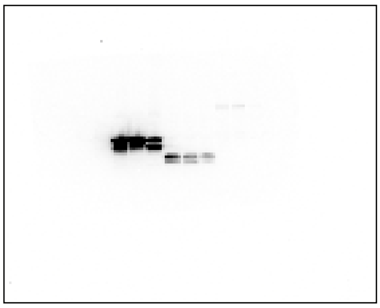

chemiluminescence-membrane overlay

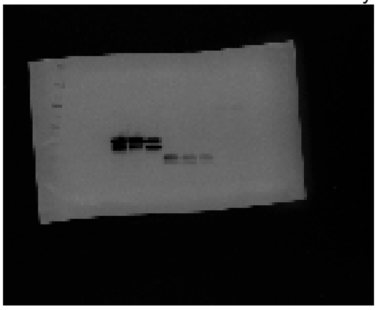

total protein

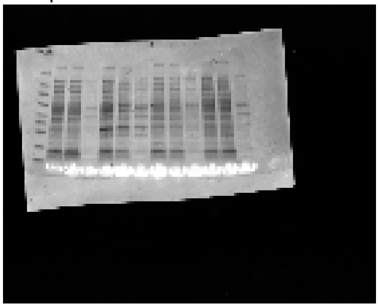

Supplement: Supplementary file 3 — Unprocessed immunoblots and gels for Extended Data Fig. 6. [file 41564_2024_1707_MOESM3_ESM.pdf]
